# Glycemic Stress Index: Does It Correlate with the Intensive Care Length of Stay?

**DOI:** 10.3390/children10020328

**Published:** 2023-02-09

**Authors:** Mathieu Georges, Thomas Engelhardt, Pablo Ingelmo, Federico Mentegazzi, Gianluca Bertolizio

**Affiliations:** 1 Department of Anesthesia, Montreal Children’s Hospital, McGill University, Montreal, QC H4A 3J1, Canada; 2 Intensive Care Medicine, Queens Hospital, BHR University Hospital, Romford RM7 0AG, UK

**Keywords:** preoperative fasting, Glycemic Stress Index, congenital heart disease, PICU length of stay

## Abstract

Postoperative hyperglycemia is an independent risk factor for postoperative complications. In adults, perioperative hyperglycemia is influenced by prolonged fasting, but data in children are lacking. The Glycemic Stress Index (GSI) has been shown to predict prolonged Pediatric Intensive Care Unit (PICU) stays in neurosurgical patients. This study aimed to confirm the correlation between GSI and duration of intubation, PICU stay, and postoperative complications in infants undergoing elective open heart surgery. The correlation between preoperative fasting and GSI was also investigated. Methods: A retrospective chart review of 85 infants ≤ 6 months undergoing elective open heart surgery was performed. GSI values ≥ 3.9 and 4.5 were tested to determine whether they carried a higher incidence of postoperative complications (metabolic uncoupling, kidney injury, ECMO, and death). The correlation between GSI and the length of intubation, PICU stay, and duration of fasting were also investigated. Perioperative factors such as age, weight, blood gas analysis, use of inotropes, and risk adjustment for congenital heart surgery were also analyzed as possible predictors. Results: GSI correlated with the duration of intubation and PICU stay. A GSI ≥ 4.5, but not 3.9, was associated with a higher incidence of metabolic uncoupling. GSI was not influenced by preoperative fasting. None of the preoperative patient factors analyzed was associated with prolonged intubation, PICU stay, or PICU complications. An abnormal creatinine before surgery increased the risk of developing acute kidney injury postoperatively. Conclusions: GSI may be valuable to predict prolonged intubation, PICU stay, and metabolic derangement in infants undergoing cardiac surgery. Fasting does not appear to affect GSI.

## 1. Introduction

Surgical stress decreases the sensitivity to insulin and promotes perioperative hyperglycemia [1], which is associated with organ dysfunction, an increased risk of infection, a prolonged hospital stay, and increased mortality [2,3,4,5]. It is also negatively associated with the rise of creatinine after cardiac surgery [6].

In the pediatric population, hyperglycemia is reported in up to 90% of all patients admitted to the Pediatric Intensive Care Unit (PICU) [3]. Children with congenital heart disease (CHD) undergoing surgical repair are particularly at risk of postoperative hyperglycemia [5,7,8], lactic acidosis, and metabolic uncoupling [9]. Up to 67% of children experience severe hyperglycemia within the first 24 h after surgery [5,7,8]. 

In adults, perioperative glucose imbalance has been prevented with the reduction of preoperative fasting [10,11,12]. In children, *npo* status often exceeds the current recommendations [13,14,15] and infants ≤ 6 months may be particularly at risk of prolonged fasting [14]. Prolonged fasting has been associated with increased child irritability, post induction hypotension, and ketones production, but the correlation with intraoperative hyperglycemia is unclear [14].

The Glycemic Stress Index (GSI) has been proposed as a new tool capable of accounting for intraoperative glycemic stress in children [16,17]. Given the problematic nature of applying adult validated indexes to measure glycemic stress and insulin resistance in pediatrics, the GSI would be of particular importance in measuring metabolic derangement in this patient population [6,18]. After major pediatric neurosurgery, GSI ≥ 3.9 and 4.5 significantly predicted PICU stay > 100 h and 200 h, respectively [16]. However, the GSI has not been validated in other surgeries.

The primary aim of this retrospective study was to evaluate the correlation between GSI and the duration of intubation and PICU length of stay in infants ≤ 6 months who were undergoing elective open-heart surgical repair for congenital cardiac disease. We also investigated the relationship between prolonged fasting and GSI.

## 2. Methods

We retrospectively reviewed the hospital charts of infants undergoing surgical repair for congenital heart disease from January 2013 to December 2017 in our hospital.

Inclusion criteria were: age ≤ 6 months, congenital heart disease, open heart surgery requiring cardiopulmonary bypass, or elective surgery.

Exclusion criteria were: age > 6 months at the time of the surgery, metabolic disorders, known preoperative hyperglycemia, patients on steroid therapy, emergency surgery, patients admitted to the hospital with an ongoing preoperative intravenous infusion, or parenteral nutrition.

The Glycemic Stress Index (GSI) was calculated as previously described [16,17]:


$$\mathrm{GSI}=\frac{\mathrm{End}-\mathrm{operative}\;\mathrm{glycemia}\;(\mathrm{mg}/\mathrm{dL})}{\mathrm{Pre}-\mathrm{operative}\;\mathrm{glycemia}\;(\mathrm{mg}/\mathrm{dL})}\times \mathrm{Surgery}\;\mathrm{duration}\;(h)$$

Preoperative glycemia was obtained from the preoperative blood work, whereas intraoperative and postoperative glycemic values, blood gas parameters, and lactate levels were obtained from the arterial blood gas analysis after the induction of anesthesia, at the end of the surgery, and at PICU admission. Within the first 24 postoperative hours of PICU admission, the highest glycemia and lactatemia values were recorded. The cutoff values of GSI ≥ 3.9 and 4.5 [16] were used to identify groups at risk of prolonged PICU stay, prolonged intubation, and PICU complications.

The length of intubation and PICU length of stay were calculated from the time of PICU admission.

To investigate a possible correlation between *npo* status and GSI, we defined prolonged fasting as ≥3 h for clear fluids, ≥5 h for breast milk, and ≥7 h for formula milk.

Medications that may affect perioperative glycemia (such as steroids and inotropes), vasopressors, diuretics, and postoperative complications (such as infections, the need for renal replacement therapy, ECMO, and death) were also noted. Hyperglycemia was defined as a glucose level greater than 6.1 mmol/L (≥110 mg/dL), whereas severe hyperglycemia was defined as a glucose level greater than 11.1 mmol/L (≥200 mg/dL) [4]. Metabolic uncoupling was defined as the simultaneous presence of severe hyperglycemia (≥11.1 mmol/L) and hyperlactatemia (≥3.5 mmol/L) [9].

Acute kidney injury was defined based on the AKIN criteria: stage 1 was defined as an increase in serum creatinine (sCr) level by 0.3 mg/dL or more and an increase of 150% to 200% of the preoperative sCr value; stage 2 was defined as an increase of 200% to 300% of the preoperative sCr value; and stage 3 was defined as an increase of >300% of the preoperative sCr value, or an sCr > 4.0 mg/dL [19]. As per institutional protocol, all patients received a dose of methylprednisolone (30 mg/kg) after the first arterial blood gas and before the surgical incision.

The primary endpoint was to correlate the GSI with the duration of intubation and the PICU length of stay. The secondary endpoints were to investigate if GSI ≥ 3.9 and 4.5 were associated with a higher incidence of postoperative complications and to correlate the fasting time with the GSI.

Based on previous data [16], a minimum of 85 patients were required to detect a significant correlation between the GSI and the length of the PICU stay with a probability (power) of 0.8. The type I error probability associated with the t-test of this null hypothesis is 0.05.

Only charts with complete information about glycemic values and PICU length of stay were considered for analysis.

Data were analyzed using SPSS software (version 21; SPSS, Inc., Chicago, IL, USA) and presented as descriptive statistics. Continuous, normally distributed variables are expressed as mean ± standard deviation, or as median + 95% confidence intervals (CI) if not normally distributed. Categorical variables are presented as percentages. Student or Mann–Whitney tests were used. A Pearson product-moment test was employed to measure the strength of the association between the outcome measures and GSI. Moreover, Pearson product-moment tests were conducted to determine whether the continuous or dichotomous independent variables were correlated with the continuous dependent variables. Independent variables that had statistically significant correlations were further evaluated using a multiple linear regression model to see if the independent variable could predict the continuous dependent variable. Multiple linear regression and logistic models were employed to determine whether perioperative factors [weight, age in months, Risk Adjustment for Congenital Heart Surgery-1 (RACHS-1), preoperative creatinine, surgical and anesthesia time, intraoperative use of inotropes or vasodilators, intraoperative lactate, and base excess] could predict the study outcomes. Proportions were compared with a Chi-square or Fisher exact test, as appropriate. A *p*-value < 0.05 was considered statistically significant.

## 3. Results and Discussion

A total of 266 charts were reviewed, and 85 children met the inclusion criteria. Patients and surgical characteristics are reported in Table 1. No data were missing.

According to the RACHS-1, 66 (77.6%) of the cases had a category 2, whereas 19 (22.4%) were classified in category 3.

**Figure 1:**
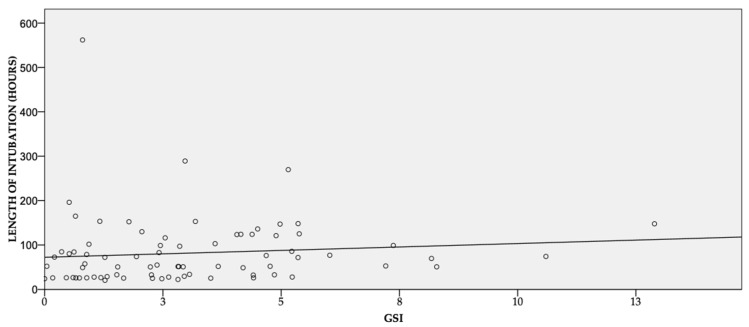
Correlation between length of intubation and GSI.

**Figure 2:**
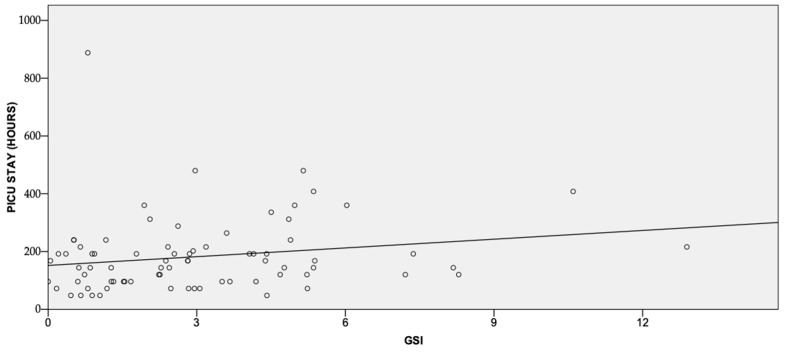
Correlation between PICU stay and GSI.

There was a weak correlation between GSI and both the length of intubation (r = 0.3, *p* = 0.014), PICU stay (r = 0.3, *p* = 0.006) (Figure 1 and Figure 2), and metabolic uncoupling at PICU admission (*p* = 0.55, *p* < 0.001).

Compared to children with a GSI < 4.5, those with a GSI ≥ 4.5 showed a longer PICU stay (median 156 h, 95% CI 132–194 vs. 144 h, 95% 163–272, *p* = 0.04) and a higher lactate at PICU admission (median 19 mmol, 95% CI 1.7–2.5 vs. 1.4 mmol, 95% 1.4–1.8, *p* = 0.01). On the contrary, the length of intubation was not statistically different (median 75 h, 95% CI 61–144, vs. 50 h, 95% 55–96, *p* = 0.09). A GSI ≥ 3.9 was not associated with any statistically significant result.

**Figure 3:**
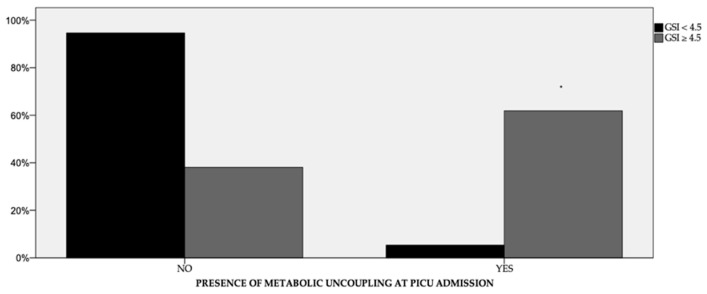
Incidence of metabolic uncoupling at PICU admission between patients with a GSI higher or lower than 4.5. The incidence of metabolic uncoupling in patients with a GSI ≥ 4.5 is statistically higher than in patients with a GSI < 4.5 (Mann–Whitney test, * *p* < 0.01).

About half of the children (49.4%) showed severe hyperglycemia at PICU admission, and 16 (18.8%) had metabolic uncoupling. Metabolic uncoupling (Figure 3) was more common in children with a GSI ≥ 4.5 than in those with a GSI < 4.5 (62% vs. 5%, *p* < 0.01) and resolved in over 95% of the patients in both groups within 24 h.

None of the preoperative conditions analyzed (weight, age in months, RACHS-1, preoperative creatinine, surgical and anesthesia time, intraoperative use of inotropes or vasodilators, intraoperative lactate, and base excess) correlated with length of intubation, PICU length of stay, or postoperative complications. We did not find any correlation between the GSI values and the rate of postoperative complications in the first 24 h. Infants with abnormal creatinine before surgery had a 28% increased risk of developing acute kidney injury postoperatively (OR 1.28, 95% CI 1.03–1.58).

Fasting time data were available for 45 patients. Twenty-seven (60%) children received oral fluids in compliance with the institutional guidelines, whereas 18 (40%) had prolonged fasting (median delay 309 min, range 61–370 min). We did not find any correlation between prolonged fasting and the GSI values.

This study showed a weak correlation between GSI, the duration of intubation, PICU stays, and the presence of metabolic uncoupling at PICU admission in infants undergoing elective open-heart surgery. Half of the patients presented with hyperglycemia at PICU admission.

It is well known that hyperglycemia is detrimental in surgical patients, including children [3,4,5]. Piastra et al. demonstrated a weak correlation (r = 0.3) between GSI and PICU stays in neurosurgical patients [16]. The authors found that a GSI ≥ 3.9 and a GSI ≥ 4.5 had high sensitivity and specificity for two different lengths of stay in the PICU. Our study confirmed not only that GSI correlates with the PICU length of stay but also with the duration of intubation. Contrary to Piastra’s results, in our study, a GSI ≥ 3.9 showed no predictability of PICU length stay, probably because our median PICU stay was shorter (144 vs. 240). Therefore, the clinical relevance of the GSI may depend on the clinical model to which it is applied, and the cutoff value used may not have a universal application.

We found no correlation between GSI and postoperative complications, except for hyperlactatemia and metabolic uncoupling. Metabolic uncoupling, defined as the simultaneous presence of hyperglycemia ≥ 11.1 mmol/L and hyperlactatemia ≥ 3.5 mmol/L, is common in pediatric cardiac surgery [9]. Metabolic uncoupling may be a transient phenomenon that does not require intervention in children with reassuring hemodynamics, or it may be a sign of systemic hypoperfusion in those patients with low cardiac output syndrome [9]. Our cohort showed a higher incidence of metabolic uncoupling compared to the literature (18.8% vs. 9.8%) [9]. In particular, patients with a GSI ≥ 4.5 had a higher incidence of metabolic uncoupling. Although patients in our cohort were not characterized by hemodynamic instability, our findings suggest that GSI may have a role in predicting this phenomenon.

With respect to the *npo* status, our incidence of patients exceeding the fasting guidelines was similar to what is reported in the literature [13,14,15]. Although in adults prolonged fasting increases insulin resistance and the incidence of intraoperative hyperglycemia [10,11,12], our study did not find any correlation between the *npo* status and the GSI. This result suggests that the GSI is not influenced by prolonged fasting.

In adults, a recent study tested different learning machine algorithms employing a number of perioperative demographic data, comorbidities, and laboratory results to successfully predict intensive care length of stay and patient mortality [20]. Platelet count, LDH, and lactate were found to be the key determinants of the study outcomes. In our investigation, the preoperative platelet count was normal in all patients, whereas LDH was not measured. In our investigation, an increased lactate level at the end of the surgery was not predictive of prolonged intubation or PICU length of stay. Machine learning has been proposed in a study to predict PICU mortality [21], but the algorithm did not include infants undergoing open heart surgery.

In children, several studies investigated whether perioperative conditions could predict PICU length of stay and patient outcome, employing different algorithms [22]. Recently, Brandi et al. [23] developed a prediction model that showed an accuracy of 71% and 69% in predicting the PICU length of stay of 3–4 and >4 days, respectively. The algorithm employed several predictions, such as age, sex, reason and type for admission, the origin of the patient (i.e., pediatric unit outpatient), patient conditions, postoperative conditions, and the Pediatric Logistic Organ Dysfunction (PELOD) score. This model, however, could not be employed in our population for several reasons. First, our cohort was limited in terms of age, type of admission (elective surgery, same-day admission), and the patient’s preoperative condition, which made most of the predictors in Brandi et al.’s study not applicable. Second, our PICU does not use scoring systems such as the PELOD or the more recent PELOD-2. In a previous investigation [24], the application of the PELOD score in children with CHD admitted to the PICU postoperatively showed a poor correlation with patient outcomes.

Children undergoing CHD surgery represent a particular population that has been often excluded from most of these studies. Jeffries et al. [25] correlated 13 predictors of children undergoing cardiac surgery with only patient mortality. On the contrary, Parkman et al. [26] investigated predictors for PICU length of stay and complications in children < 1 year of age undergoing cardiac surgery. The authors found that weight significantly predicted the hospital length of stay in 46% of the cases, whereas age and comorbidities correlated with the incidence of postoperative complications. The authors did not investigate the influence of perioperative glycemia on patient outcomes. Compared to Parkman et al.’s study, our cohort had a narrower age range (<6 months), was healthier (outpatients), and cardiac operations were less complex (RACHS-1 category 2 and 3), which may explain the different results.

## 4. Limitation of the Study

Our study has important limitations that must be considered. First of all, it is single center, retrospective study, which inherently carries a bias in patients’ selection.

Secondly, we limited our investigation to outpatients undergoing elective open-heart surgery. This choice was dictated by the need to collect the *npo* status of our cohort. Patients admitted to the hospital do, in fact, have intravenous fluid administration preoperatively, which would impair the definition of fasting.

Moreover, we could not collect the intraoperative glycemic trend; therefore, we cannot exclude that prolonged fasting transiently influences intraoperative glycemia, although it appears that the magnitude of this effect, if any, is not significant enough to affect the GSI.

Lastly, the spectrum of CHD includes a wide range of malformations, associated comorbidities, and surgical options. As a consequence, both patient conditions and surgical stress vary widely, and this may affect the validity of the GSI.

## 5. Conclusions

The GSI may be a valuable tool to predict metabolic derangement and identifying patients who will require a long PICU stay. However, it has only been validated in selected surgical patients, and further studies are needed to confirm its usefulness and cutoff value in more complex patients, such as those with low cardiac output syndrome. In stable infants undergoing moderately complex open-heart surgery, GSI may be a valuable predictor to be included in future machine learning or other prediction models. The GSI appears not to be influenced by the fasting status of the patient.

## Figures and Tables

**Table 1:** Demographic and surgical data.

Demographic Data (Mean ± SD)	
Age (months)	5.1 (± 0.9)
Weight (kg)	35 (± 1.5)
Operation variables (median, 95% CI)	
Length of surgery (min)	226 (217–240)
Length of anesthesia (min)	350 (347–380)
Length of bypass (min)	118 (112–128)
Length of cross-clamp (min)	67 (65–76)
Type of CHD (n, %)	
Aortic stenosis	1 (1.2)
Atrial septal defect	1 (1.2)
Atrioventricular septal defect	10 (11.8)
Double outlet right ventricle	7 (8.2)
Pulmonary stenosis	4 (4.7)
Pulmonary venous stenosis	1 (1.2)
Tetralogy of Fallot	21 (24.7)
Ventricular septal defect	40 (47.1)
Type of surgery (n, %)	
Atrial septal defect closure	1 (1.2)
Aortic valve repair	1 (1.2)
Atrioventricular septal defect repair	10 (11.8)
Double outlet right ventricle repair	7 (8.2)
Pulmonary artery repair	3 (3.5)
Pulmonary venous stenosis repair	1 (1.2)
Pulmonary valve repair	1 (1.2)
Tetralogy of Fallot repair	21 (24.7)
Ventricular septal defect repair	40 (47.1)
Preoperative medications (n, %)	
Diuretics	43 (50.6)
Beta blockers	6 (7.1)
Digoxin	1 (1.2)
Others (i.e., H2 antagonist, PPI, iron)	10 (41.1)
Intraoperative medications (n, %)	
Epinephrine	41 (48.2)
Dopamine	60 (70.6)
Dobutamine	7 (8.20)
Milrinone	79 (92.9)
Nitroglycerine	1 (1.2)
Nitroprusside	3 (3.5)
Esmolol	5 (5.9)

## Data Availability

Data are available upon request to the corresponding author.
